# Evaluating the Role of GPT-4 and GPT-4o in the Detectability of Chest Radiography Reports Requiring Further Assessment

**DOI:** 10.7759/cureus.75532

**Published:** 2024-12-11

**Authors:** Jun Kanzawa, Ryo Kurokawa, Masafumi Kaiume, Yuta Nakamura, Mariko Kurokawa, Yuki Sonoda, Wataru Gonoi, Osamu Abe

**Affiliations:** 1 Radiology, University of Tokyo, Tokyo, JPN

**Keywords:** chest radiography, gpt-4, gpt-4o, large language model, radiology

## Abstract

Purpose

The aim of this study is to investigate the capability of generative pre-trained transformer 4 (GPT-4) and GPT-4o in identifying chest radiography reports requiring further assessment.

Materials and methods

This retrospective study included 100 cases from the National Institutes of Health chest radiography dataset, including 50 abnormal and 50 normal cases. A radiologist blinded to the study's purpose interpreted and reported the radiological findings for each case in English and separately determined the necessity for further assessment based on predefined criteria as referential standards. The radiology reports were then input into GPT-4 and GPT-4o models, accompanied by a prompt to identify cases requiring further assessment. This procedure was repeated five times in separate sessions for each model. Overall accuracy, sensitivity, and specificity of the necessity for further assessment were assessed using McNemar’s test. Positive and negative predictive values were assessed using Fisher’s exact test and Chi-square test, respectively.

Results

A total of 100 cases were included (mean age of 49.4 years ± 15.4 [standard deviation]; 56 women). Among them, 44 were judged by the radiologist to require further assessment. Across the five sessions, 19.6% and 35.8% of the cases were judged to require further assessment by GPT-4 and GPT-4o, respectively. The sensitivity, accuracy, and negative predictive value of GPT-4o (74.5%, 85.8%, and 82.6%, respectively) were all significantly higher than those of GPT-4 (44.5%, 75.6%, and 69.7%, respectively) (*p* < 0.001). The specificity and positive predictive value of GPT-4 (100% and 100%, respectively) were significantly higher than that of GPT-4o (94.6% and 91.6%, respectively) (*p* < 0.001).

Conclusion

GPT-4o showed acceptable performance in detecting chest radiography reports requiring further assessment.

## Introduction

Significant research has focused on automatically detecting actionable radiology reports, preventing clinicians from overlooking critical information and improving patient management [1]. Recent advancements in natural language processing (NLP) have made it possible to detect necessary information from radiology reports in free-text format [2].

Chest radiography is one of the frequently studied targets for NLP applications [2], as it is commonly performed for routine check-ups and screening purposes, even in asymptomatic patients [3]. These radiographic images often contain findings that may not directly relate to the patient's presenting symptoms [4], which implies that manual inspection of all radiology reports could delay the provision of care to those who need it most. By automatically extracting chest radiography reports containing clinically significant findings, clinicians can determine cases that require immediate attention and improve clinical practice efficiency. It can also help improve patient safety by automatically alerting clinicians to critical information in reports. Previous studies have reported on the automatic extraction of conditions such as acute lung injury [5], pneumonia [6], pneumothorax [7], and misplaced medical devices [7]. These studies employed various NLP techniques, including keywords, lexicons, and machine learning-based algorithms [5,6]. However, previous studies focused on detecting a few specific conditions. In addition, these studies often involved technical challenges, such as the need to develop complex models and train them with extensive datasets. As a result, the existing approaches are difficult to implement in broad facilities for a wide range of purposes.

The recent advancement in large language models (LLMs), particularly the introduction of generative pre-trained transformer 4 (GPT-4) [8], has greatly enhanced the capabilities of NLP [9]. Several studies have investigated the potential of utilizing GPT-4 for various radiology tasks without extensive training data [9]. For instance, researchers have applied GPT-4 to generate the impression section of radiology reports based on the findings section [10], automatically determine appropriate study protocols [11], and convert free text reports into structured format [12]. In addition to the clinical application, GPT-4 also covered considerable information on radiology, exemplified by its proficiency in answering radiological case quizzes [13].

Despite the promising applications of GPT-4 in radiology, its capability to accurately identify actionable radiology reports remains to be explored. This question is important as it determines whether GPT models can effectively support clinical decision-making by non-radiologist physicians.

This study aims to investigate the capability of GPT-4 and its latest model, GPT-4o [14], in identifying chest radiography reports requiring further assessment.

## Materials and methods

The requirement for obtaining written informed consent from patients was waived due to the use of publicly available data. From the National Institutes of Health chest radiography dataset [15], 50 images labeled as “No Finding” and 50 images with any type of abnormality labels were selected using randomized subject ID numbers. In cases in which a patient had multiple follow-up images, the initial image was chosen for the study. Criteria for classifying chest radiography findings into those requiring further assessment or not were made based on previous studies, as shown in Table 1 [3,4].

**Table 1:** Criteria to classify chest radiography findings

Findings requiring further assessment	Findings not requiring further assessment
Nodular shadows or infiltrates	Calcifications (aorta, blood vessels, lymph nodes)
Aortic dilatation	Scoliosis, pectus excavatum
Enlargement of the hilum or mediastinum	Degenerative changes of the spine, old rib fractures
Interstitial lung disease	Elongation or tortuosity of the aorta
Vertebral compression fractures	Pleural or pulmonary scars: adhesions, granulomas, atelectasis, etc.
Pleural effusion	Other: situs inversus, supernumerary ribs, breast implants, etc.
Cardiomegaly or pulmonary congestion	Pacemaker
Findings suggestive of chronic obstructive pulmonary disease or asthma	Technical issues: insufficient inspiration, overlapping breast tissue, patient rotation, etc.
	Hiatal hernia

A board-certified radiologist (M.K.) with eight years of imaging experience, who was blinded to the purpose of this study, interpreted the images and wrote reports in English for each image and determined whether further assessment was necessary (positive group) or not (negative group) based on the aforementioned criteria for each case. If there was a finding not included in the criteria, the necessity of assessment was determined based on the radiologist’s opinion in agreement with another board-certified radiologist (R.K.) with 10 years of imaging experience. Another radiologist (J.K.) with three years of imaging experience screened the reports to ensure that they did not contain any explicit statements indicating the necessity of further assessment. The reports were then input into both GPT-4 [8] and GPT-4o [14] models, accompanied by a prompt: “Based on the chest radiography reports, please identify cases that require further assessment.” The classification criteria were not presented to the models. The same prompt was posed five times to account for the variation in responses from LLMs every time [16]. Each evaluation was conducted in separate chat sessions to ensure that GPT's responses were not influenced by previous interactions, as the model's memory is completely deleted between sessions. Overall accuracy and sensitivity were compared between GPT-4 and GPT-4o using McNemar’s test. Specificity was compared using exact McNemar’s test. Positive predictive value (PPV) and negative predictive value (NPV) were compared between GPT-4 and GPT-4o using Fisher’s exact test and chi-square test, respectively. Statistical analyses were performed using R version 4.3.1 (R Core Team, R Foundation for Statistical Computing, Vienna, Austria, https://www.r-project.org/). Statistical significance was set at a p-value of < 0.01.

## Results

Table 2 summarizes the demographic details of the dataset. The positive group consisted of 44 cases (mean age of 52.3 years ± 18.0; 26 women) and the negative group consisted of 56 cases (mean age of 47.0 years ± 12.5; 33 men). There was no disagreement between the two radiologists on case categorization.

**Table 2:** Demographic details of the dataset The positive group indicates those requiring further assessment. The negative group indicates those not requiring further assessment.

	Positive group	Negative group
Number of reports	44	56
Age (mean ± standard deviation)	52.3 ± 18.0	47.1 ± 12.5
Sex (male/female)	18/26	33/23

Table 3 summarizes the main findings in chest radiography reports of each group.

**Table 3:** Main findings in chest radiography reports The positive group indicates those requiring further assessment. The negative group indicates those not requiring further assessment.

Findings	Number of cases
Positive group (44 cases)	
Nodule or mass	16
Pleural effusion	8
Cardiomegaly	6
Consolidation or ground-glass opacities	5
Elevated diaphragm	3
Interstitial changes	2
Pneumothorax	2
Enlargement of mediastinum	1
Hyperinflation	1
Negative group (56 cases)	
No findings	40
Postoperative changes	7
Peripherally inserted central venous catheter or central venous port	6
Pleural adhesion	1
Tortuosity of the aorta	1
Dextrocardia	1

Table 4 shows the confusion matrix for the reference standard and answers by GPT-4 and GPT-4o. A total of 100 cases were evaluated five times by each model, resulting in 500 total classifications per model. The confusion matrix summarizes these 500 evaluations.

**Table 4:** Confusion matrix for the reference standard versus answers of large language models The data represent the total number of classifications evaluated by the models. The positive group indicates those requiring further assessment. The negative group indicates those not requiring further assessment.

	Reference standard	
	Positive group (220 cases)	Negative group (280 cases)
GPT-4		
Positive (98 cases)	98	0
Negative (402 cases)	122	280
GPT-4o		
Positive (179 cases)	164	15
Negative (321 cases)	56	265

Table 5 demonstrates the performance metrics of the models. GPT-4o showed significantly improved performance in sensitivity, specificity, accuracy, PPV, and NPV compared with GPT-4.

**Table 5:** Performance metrics of GPT-4 and GPT-4o in identifying chest radiography reports requiring further assessment PPV, positive predictive value; NPV, negative predictive value Numbers in parentheses represent the numerator and denominator for each calculation. Data in brackets represent the 95% confidence intervals. Comparisons were performed using McNemar’s test for sensitivity and accuracy and exact McNemar’s test for specificity. Fisher’s exact test and chi-square test were used for PPV and NPV, respectively. Test statistics represent chi-square values for sensitivity, accuracy, and NPV. A p-value of <0.01 is considered statistically significant.

	GPT-4			GPT-4o			Test statistic	Comparisons (p)
Sensitivity (%)	44.5	(98/220)	[37.9–51.4]	74.5	(164/220)	[68.3–80.2]	44.0	<0.001
Specificity (%)	100	(280/280)	[98.7–100]	94.6	(265/280)	[91.3–97.0)]		<0.001
Accuracy (%)	75.6	(378/500)	[71.6–79.3]	85.8	(429/500)	[82.4–88.7]	57.7	<0.001
PPV (%)	100	(98/98)	[96.3–100]	91.6	(164/179)	[86.6–95.2]		<0.001
NPV (%)	69.7	(280/402)	[64.9–74.1]	82.6	(265/321)	[78.0–86.5]	15.3	<0.001

Across the five sessions, 19.6% and 35.8% were judged to require further assessment by GPT-4 and GPT-4o, respectively. GPT-4o demonstrated a sensitivity of 74.5% (164/220, 95% CI: 68.3-80.2), specificity of 94.6% (265/280, 95% CI: 91.3-97.0), accuracy of 85.8% (429/500, 95% CI: 82.4-88.7), PPV of 91.6% (164/179, 95% CI: 86.6-95.2), and NPV of 82.6% (265/321, 95% CI: 78.0-86.5). GPT-4 demonstrated a sensitivity of 44.5 (98/220, 95% CI: 37.9-51.4), specificity of 100% (280/280, 95% CI: 98.7-100), accuracy of 75.6 (378/500, 95% CI: 71.6-79.3), PPV of 100% (98/98, 95% CI: 96.3-100), and NPV of 69.7 (280/402, 95% CI: 64.9-74.1).

The sensitivity, accuracy, and NPV were significantly higher in GPT-4o than in GPT-4 (p < 0.001). The specificity and PPV were significantly higher in GPT-4 (p < 0.001).

## Discussion

Our study demonstrated that GPT-4o could identify chest radiography reports that require further assessment with higher accuracy, sensitivity, and NPV compared to GPT-4 (p<.001). Although GPT-4o demonstrated lower specificity and PPV compared to GPT-4, it still achieved a high specificity of 94.6% and PPV of 91.6%. These results highlight the promising application of the latest LLMs in detecting chest radiography reports warranting further assessment.

One previous study used the maximum entropy algorithm with character 6-gram to classify acute lung injury chest X-ray reports, achieving recall=0.91, precision=0.90, and F-measure=0.91, outperforming the keyword-based system and comparable to the highest performing physician annotators [5]. Another study developed a rule-based NLP system in classifying chest radiography reports as either negative or possible/positive for pneumonia, achieving a sensitivity of 92.7%, specificity of 91.1%, PPV of 93.3%, and NPV of 90.3% [6]. In addition, fine-tuned bidirectional encoder representations from transformers (BERT) model demonstrated notable performance in extracting information from free-text medical reports, with areas under the receiver operating characteristic curve of 0.98 for congestion, 0.97 for effusion, 0.97 for consolidation, and 0.99 for pneumothorax [7]. The current study expands on these previous findings by demonstrating that GPT-4o can detect cases requiring further assessment with various types of abnormalities including nodule, consolidation, and interstitial changes, making it easier to apply in real clinical settings. Considering the improvement in performance from GPT-4 to GPT-4o, further enhancements can be anticipated with future updates.

Although superior to GPT-4, sensitivity of GPT-4o was not as high as other metrics. Specifically, across five sessions, two cases of elevated diaphragm and a case of hyperinflation were not correctly judged as requiring further assessment. It is also important to note that the sensitivity of GPT was partly affected by inconsistency in answers across sessions. A concrete example of where these limitations might impact clinical outcomes is as follows: since the sensitivity of LLM is not perfect, clinicians would still need to manually review all reports to prevent oversight of critical findings. This implies that LLM cannot fully automate the process or drastically reduce the clinicians' workload, especially in high-volume clinical settings. Further evaluation is needed to determine whether sensitivity could be improved through model updates or other approaches, such as optimizing prompt designs, as high sensitivity is crucial in screening settings.

Notably, in contrast to existing literature, GPT was not given any criteria regarding which findings to focus on when determining clinical significance. Furthermore, the model was not provided with explicit indications for further assessment within the reports. Given the ability of GPT-4o to independently analyze radiology reports, it can serve as an effective tool for clinicians by providing support in the clinical decision-making process. This can eventually contribute to patient safety by promoting timely assessment of the critical findings identified in reports.

Another difference with existing NLP approaches is that GPT-4o exhibited satisfactory performance without additional training on a massive dataset. For example, a previous study utilizing BERT required extensive training on large datasets of radiology reports, involving multiple training steps including pre-training and fine-tuning [7]. The absence of such complex training requirements suggests that our approach is generalizable to other settings, despite potential differences in radiology report formats.

Although it is possible that open-access data were used in the GPT model training, our study did not use open-access images directly. Instead, we analyzed original radiology reports written by radiologists for these images. This method ensured that the GPT model did not rely on existing knowledge. Although GPT model training may include open access data, our study did not use open-access images directly. Instead, we analyzed original radiology reports written by radiologists at our institution. These reports were not part of GPT's training data, as they have never been publicly released. This ensures that the model's assessments were not based on existing knowledge of these specific cases. Therefore, the potential use of open-access data in GPT's training does not limit the validity of our assessment. Rather, the use of radiology reports based on open-access data is beneficial as it facilitates the reproducibility of research findings.

The present study has several limitations such as a relatively small number of cases, restriction to chest radiographs, and a prevalence of abnormality that does not reflect clinical practice. More specifically, the small number of cases could impact external validity, as it is impossible to encompass all possible etiologies seen on chest radiography in a single study. In addition, real patient data were not evaluated due to privacy concerns. Although the simplicity of our approach would likely be readily applicable to other settings, further investigation is warranted in larger cohorts and other modalities to validate our findings in real clinical practice. The black box nature of LLMs and potential bias in model training raise concerns about how much clinicians can rely on LLMs' decisions. Regarding ethical considerations, the deployment of LLMs in clinical practice presents ethical problems regarding patient data transmission to third-party providers and potential use of this data in future model training. Standard guidelines are necessary to establish how clinicians should utilize LLMs in clinical decision-making and ensure patient privacy protection.

Although comparing multiple LLMs would expand the findings, this study focused on GPT models for several reasons. First, this study represents, to our knowledge, the first investigation of using LLMs for radiology report classification. Second, GPT models have been extensively validated across various radiological applications, including the generation of radiology report impression section [10], automated determination of study protocols [11], and structured report construction [12]. The approach of these previous studies is similar in that they focused primarily on evaluating GPT models' performance. Given GPT models' extensive validation in radiology and their effectiveness across various radiological applications, we determined that utilizing GPT was appropriate for establishing baseline performance of LLM in radiology report classification. Future studies could expand upon our findings by evaluating other LLMs.

## Conclusions

GPT-4o showed improved performance in detecting chest radiography reports requiring further assessment compared to GPT-4. Compared to existing approaches, the use of GPT for radiology report classification is efficient as it eliminates the need for complex model development. The results highlight the promising application of the latest LLM in assisting with clinical decision-making based on imaging findings, especially useful for clinicians involved in primary care or emergency medicine. Future studies should focus on validating these findings in diverse clinical settings and various imaging modalities.
